# Molecular Chaperones of *Leishmania*: Central Players in Many Stress-Related and -Unrelated Physiological Processes

**DOI:** 10.1155/2015/301326

**Published:** 2015-06-18

**Authors:** Jose M. Requena, Ana M. Montalvo, Jorge Fraga

**Affiliations:** ^1^Centro de Biología Molecular “Severo Ochoa” (CSIC-UAM), Universidad Autónoma de Madrid, 28049 Madrid, Spain; ^2^Departamento de Parasitología, Instituto de Medicina Tropical “Pedro Kourí”, 17100 Habana, Cuba

## Abstract

Molecular chaperones are key components in the maintenance of cellular homeostasis and survival, not only during stress but also under optimal growth conditions. Folding of nascent polypeptides is supported by molecular chaperones, which avoid the formation of aggregates by preventing nonspecific interactions and aid, when necessary, the translocation of proteins to their correct intracellular localization. Furthermore, when proteins are damaged, molecular chaperones may also facilitate their refolding or, in the case of irreparable proteins, their removal by the protein degradation machinery of the cell. During their digenetic lifestyle, *Leishmania* parasites encounter and adapt to harsh environmental conditions, such as nutrient deficiency, hypoxia, oxidative stress, changing pH, and shifts in temperature; all these factors are potential triggers of cellular stress. We summarize here our current knowledge on the main types of molecular chaperones in *Leishmania* and their functions. Among them, heat shock proteins play important roles in adaptation and survival of this parasite against temperature changes associated with its passage from the poikilothermic insect vector to the warm-blooded vertebrate host. The study of structural features and the function of chaperones in *Leishmania* biology is providing opportunities (and challenges) for drug discovery and improving of current treatments against leishmaniasis.

## 1. The Stressful Life of* Leishmania* Parasites

There is growing body of evidence that chaperones play important roles in the life cycle of a wide variety of important human pathogens [1, 2]. In particular, the life cycle of insect-transmitted parasites entails multiple changes in environmental conditions, including temperature, pH, oxidative stress, and nutrient deprivation. These stressful conditions may damage important cellular structures and interfere with essential functions of the parasites.

Parasites of the genus* Leishmania* cause leishmaniasis in humans and in a variety of vertebrate hosts. In humans, leishmaniasis constitutes a group of diverse diseases ranging in severity from a spontaneously healing skin ulcer to overwhelming visceral disease [3]. Above 350 million people are currently at risk of acquiring the various forms of the disease, and it was estimated that approximately 0.2 to 0.4 million of visceral leishmaniasis (VL) cases and 0.7 to 1.2 million cutaneous leishmaniasis (CL) cases occur each year [4]. CL has a spectrum of presentations, typically with self-healing or chronic lesions on the skin. In some cases, after resolution of the initial cutaneous lesion, metastasis of the parasite occurs and tissue destruction appear on the buccal or nasal mucosa, process known as mucocutaneous leishmaniasis (MCL).

Around 20* Leishmania* species are pathogenic for humans, and they are spread by the bite of an infected sandfly (phlebotomines of the genera* Phlebotomus*,* Lutzomyia*, and* Psychodopygus*), which transmits the promastigote stage of the parasite into the human skin. Promastigotes are phagocytosed by macrophages, either directly or after initial uptake by neutrophils, and are delivered to the mature phagolysosome compartment where they differentiate to the nonmotile amastigote stage. Thus,* Leishmania* spp. undergo a complex life cycle involving progression from the insect vector (promastigote stage, with an anterior flagellum) to a mammalian host (amastigote stage with only a very short flagellum) and back again [5]. During its life cycle, the parasite continually cycles between a poikilothermic insect vector and a warm-blooded vertebrate host and therefore experiences repeated episodes of heat shock and oxidative stress, among other environmental changes [6].

The aim of this review is to survey the molecular chaperone compendium in this parasitic protist and what is known about how these molecules contribute to alleviate the molecular consequences of the stressful environmental conditions that the parasite must cope in its intracellular and extracellular lifestyles. Beyond their roles for cytoprotection against stress, parasite heat shock proteins (HSPs) have been implicated in differentiation, stage development, and virulence. Although many chaperones have been shown to elicit strong immunological responses in infected individuals, their usefulness as potential vaccine candidates has been reviewed elsewhere [7] and this issue will not be discussed in this review.

## 2. Molecular Chaperones

In the cell, at any given time, hundreds of macromolecular processes involving proteins are occurring. In the cytosol, proteins are present at extremely high concentration (over 300 mg/mL); therefore, protein-protein interactions must be governed and modulated appropriately [9]. The modern concept of protein quality control refers to those cellular processes that regulate protein synthesis, folding, unfolding, and turnover, and it is mediated by chaperone and protease systems. The quality control systems have essential roles in the life of cells, ensuring that proteins are correctly folded and functional at the right place and time. Molecular chaperone is the term used for referring to any protein that shares the ability to interact with nonnative conformations of other proteins. Molecular chaperones aid in the folding of nascent polypeptides as they are synthesized by ribosomes, transit across cellular and organelle membranes, disassembly of macromolecular complexes or aggregates, and quality control processes, as well as the regulation of conformational changes that affect biological functions of proteins [10, 11]. More recently, the term chaperome has been coined for the interconnected network of molecular chaperones as well as cochaperones that assist in their function [12, 13]. The chaperones have the ability to distinguish between unfolded, misfolded, and native protein conformers. Of particular importance is the shielding of hydrophobic residues, which are temporally exposed during initial folding but also upon damaging of existing proteins. Uncontrolled exposure of hydrophobic stretches leads to protein aggregation and has fatal consequences. Hence, chaperones bind to exposed hydrophobic segments of substrate proteins (often named “client” proteins) with the objective of preventing the formation of stable, irreversible protein aggregates, thereby facilitating their appropriate folding. In addition to preventing aggregation, some chaperones are endowed with ATPase activity and use the energy of ATP hydrolysis to actively unfold protein substrates in order to accelerate the conversion of stable misfolded structures into stable native conformations.

Both extra- and intracellular stresses have a deleterious impact on protein structure and function. In order to counteract these effects, cells have developed the stress response, in which molecular chaperones are the central components. In fact, because many molecular chaperones were first identified as being induced by heat shock or other stresses, they are known as heat shock proteins or stress proteins [14]. The predominant classes were grouped according to their apparent molecular weights: small HSPs (sHSPs), HSP60, HSP70, HSP90, and HSP100.  Table 1 summarizes the main families of molecular chaperones. These families of chaperones are ubiquitous and conserved in most organisms, from bacteria to higher eukaryotes.

However, our understanding of the roles played by molecular chaperones in human parasites is lagging behind, although important progress has been made recently and warrants this review. Our main goal was to present the composition of the different chaperone families in* Leishmania*, by mining genome databases (the* L. major* genome annotation has been chosen as reference), and describing their known functions, by reviewing the scientific literature. Thus, our final aim is to provide a framework that may facilitate and stimulate the study of the implications of these proteins in the biology of this human pathogen that causes fearsome diseases.

## 3. HSP100/Clp Family

Protein aggregates are detrimental to the cell; therefore, living systems possess a plethora of molecular chaperones dedicated to avoid protein aggregation. In case this fails, there are two ways to solve the problem: destruction of the aggregates by the action of elaborate protease systems or nondestructive disaggregation. Chaperones of the HSP100 family has the unique capability of recognizing misfolded proteins within an aggregate and actively unfolding them, ultimately disassembling the insoluble structure and delivering substrates into refolding pathways. Giving this peculiar ability, that is, to pull protein from aggregates, they have been named as “disaggregates” [17]. For this formidable task, that is, the nondestructive disaggregation of protein aggregates, HSP100 chaperones work in combination with the HSP70/DnaK chaperone machinery. Also, in the task of avoiding irreversible protein aggregation, the role played by sHSPs seems to be very relevant [18].

The HSP100/Clp family of chaperones belongs to the superfamily of AAA+ domain-containing ATPases. The defining feature of the superfamily is the AAA+ domain, which consists of an *α*–*β* subdomain and a smaller, helical subdomain [19]. Most AAA+ proteins form oligomeric ring structures with ATP binding site close to the interface between subunits, and the neighboring subunit contributes the so-called arginine finger for ATP hydrolysis [20]. HSP100 proteins are subdivided into two classes based on the number of AAA+ domains. Class 1 HSP100s form hexameric structures and contain two different nucleotide-binding sites in each monomer [21]. Bacterial proteins ClpA, ClpB, ClpC, and ClpE as well as yeast HSP104 belong to this class. Class 2 HSP100s contain only one nucleotide binding module; examples are the bacterial ClpX and ClpY, and the AAA+ ATPase p97/Cdc48 in mammals [22]. HSP100 proteins are thought to pull misfolded proteins through the central pore of the hexameric ring, enabling the proteins to become refolded [23].

On the other hand, HSP104 is one of the major heat shock proteins that contribute to acquire thermotolerance [10]. Notably HSP104 is also involved in yeast prion inheritance [24].

An orthologue to the yeast HSP104 gene was cloned in* L. major* (systematic name LmjF29.1270); the encoded protein was named HSP100 because of its apparent molecular weight and the fact that its expression increased dramatically after incubation of* Leishmania* promastigotes at elevated temperatures [25]. The HSP100 has been found to be expressed preferentially in amastigotes, whereas it was found to be dispensable in the promastigote stage [26]. However, although an initial delay in lesion development was observed in BALB/c mice experimentally infected with an* L. major* Δhsp100 mutant, finally the lesions reached a similar size to those caused by the* L. major* wild-type line. This argues against a requirement of HSP100 for the intracellular proliferation of fully differentiated amastigotes and suggests that the loss of HSP100 chiefly affects processes in the initial phase of an infection, that is, development from the promastigote to the amastigote stage [26]. In fact, after repeated mouse infection cycles, an escape variant with restored infectivity and pathogenicity was obtained [27].

In* L. donovani*, the expression of HSP100 is required for normal development of the parasite inside mammalian host cells but it is dispensable for axenic growth. Also, it has been postulated that HSP100 may act as an antagonist for the amastigote-to-promastigote differentiation process [28].

*Leishmania* HSP100 has been found to play a very relevant function in exosome biogenesis. Thus, proteomic analysis showed that exosomes from the wild-type and a Δhsp100* L. donovani* mutant have distinct protein cargo, suggesting that packaging of proteins into exosomes is dependent in part on HSP100. Furthermore, it was found that exosomes from wild-type* L. donovani* failed to prime monocyte-derived dendritic cells to drive the differentiation of naive CD4^+^ T cells into IFN-*γ*–producing Th1 cells, whereas exosomes derived from the Δhsp100 mutant line promoted the differentiation of naive CD4^+^ lymphocytes into Th1 cells [29]. These findings demonstrate that* Leishmania* exosomes are predominantly immunosuppressive, and it can be postulated that HSP100 is modulating the composition of the exosomal cargo.

In the* L. major* genome, there are two identical genes (LmjF02.0710 and LmjF27.2630), encoding for a protein (annotated as HSP78) that is more similar to the* E. coli* ClpB protein than to the* Leishmania* HSP100 [15]. It is possible that HSP78 locates at the mitochondria (or kinetoplast) of* Leishmania*, but this has not been experimentally addressed. Also, in the* L. major* database, there is another gene (LmjF.30.1700) coding for a predicted protein with a region, comprising amino acids 324–473, that conforms the typical ATPase domain of the AAA+ superfamily.

Putative class 2 HSP100s in* Leishmania* are encoded by genes LmjF.15.0090 and LmjF.09.0230, which show remarkable sequence conservation with* E. coli* ClpY. In* E. coli*, ClpY (HslU) and ClpQ (HslV) are heat shock proteins involved in the proteolysis of misfolded proteins; both proteins form a complex known as either HslVU or ClpYQ [30]. The protein encoded by gene LmjF.15.0090 is annotated as HslU1 and that encoded by gene LmjF.09.0230 as HslU2, due to the fact that the orthologous proteins have been studied in the related trypanosomatid* Trypanosoma brucei* [31]. The* L. major* HslV is encoded by gene LmjF36.3990. By epitope tagging, TbHslVU proteins have been localized into the* T. brucei* mitochondria, where they are associated with the mitochondrial genome, kinetoplast DNA (kDNA). Of particular note is the finding that silencing by RNAi of the corresponding genes dramatically affects the kDNA replication and segregation [31]. Recently, the relevance of HslU proteins has been addressed in* L. donovani* [32]. It was found that the loss of HslU2 does not impair viability, even though a reduced growth of the promastigote stage was observed. However, the attempts to create HslU1 null-mutants failed, and the loss of a single allele resulted in reduced growth and unusual morphology, suggesting a vital role for this protein. Furthermore, results shown in this study argued against an association between HslV and the HslU proteins in* L. donovani* [32]. However, these findings have been questioned in a recent report, in which the mitochondrial localization of proteins HslU1, HslU2, and HslV in* L. major* procyclic promastigotes has been definitively established [33].

## 4. HSP90 Family

HSP90s are among the most abundant proteins, even in unstressed cells. Members of this family are found in the cytoplasm, endoplasmic reticulum (ER), and mitochondria, and they are named differently regarding their subcellular location: HSP90 (often referred as HSP83) for the cytoplasmic form, Grp94 (gp96 or endoplasmin) for the ER form, and TRAP1 (or HSP75), which is located in the mitochondrial matrix. Although HSP90 can bind to misfolded proteins and prevent their aggregation, it is accepted that HSP90s do not act in protein folding as general chaperones. More often, they are required for the activation and/or stabilization in a native state of a defined set of proteins, known as “clients.” Many of these clients are transcription factors and protein kinases involved in the control of cell homeostasis, proliferation, differentiation, and apoptosis [34, 35]. Since HSP90 clients are hubs of diverse signaling networks and participate in nearly every cellular function, HSP90s represent connectors for many regulatory circuits and link them to environmental impacts [36].

On the other hand, HSP90 acts as a buffer for genetic variation by rescuing mutated proteins with altered properties. Such a reservoir function has been suggested to be extremely relevant in driving evolutionary changes [37]. Support for this hypothesis has been obtained in studies investigating the developmental effects of HSP90 inhibitors on* Drosophila melanogaster* and* Arabidopsis thaliana*, and from studies examining the effects of environmental stresses in yeast [38].

The functional HSP90 chaperone is a flexible dimeric protein composed of three functional domains: the N-terminal nucleotide-binding domain (NBD); the middle domain (MD), which is also involved in ATP hydrolysis and it is the site for client and cochaperone binding; and a C-terminal dimerization domain (DD). A conserved MEEVD motif at the C-terminal end serves as the docking site for the interaction with cochaperones which contain the tetratricopeptide repeat- (TPR-) domain. Some cochaperones and client proteins interact with HSP90 through its NBD domain [39]. The NBD is also the binding site for the HSP90-specific inhibitors geldanamycin and radicicol [40]. The domain interfaces between NBD and MD and between MD and DD are dynamic, resulting in an ensemble of conformations.

**Figure 1:**
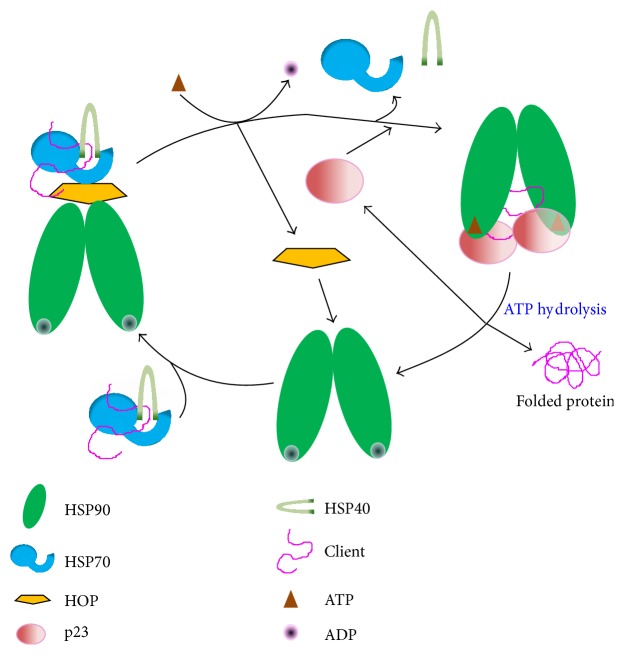
Overview of the HSP90 chaperone cycle. In the initial step, protein substrate bound to the HSP70-HSP40 chaperones interacts with HSP90; the formation of this complex is induced by HOP. ATP binding to HSP90 induces a conformation change in the complex, which leads to the substrate transfer from HSP70 to HSP90, and the release of the HSP70-HSP40 chaperones; the substrate-HSP90 complex is stabilized by p23 binding. Finally, the hydrolysis of ATP induces additional conformation changes leading to substrate release.

In eukaryotic cells, the ATPase cycle of HSP90 is intimately coupled to the HSP70 chaperone machinery (Figure 1). In fact, the emerging model is that client proteins do not bind directly to HSP90 but instead the client-substrates are firstly bound by HSP70, which control substrate influx to HSP90 [41]. Moreover, to achieve its function, HSP90 requires the participation of different cofactors (termed cochaperones) to facilitate the maturation of client proteins. To date, more than 20 cochaperones have been identified; they regulate the activity of HSP90 and determine the interactions with specific client proteins [10, 42]. Thus, the composition of cochaperone complexes seems to depend on the particular client protein that is being modulated by the HSP90 heterocomplex. HSP90 is also regulated posttranslationally through chemical modifications, including phosphorylation, acetylation, methylation, and S-nitrosylation, and even ubiquitination has been reported [36, 43, 44].

First work describing the presence of the HSP90 gene in* Leishmania* and* T. brucei* was done by Van der Ploeg and coworkers [45]. Afterwards, research from many other groups allowed the characterization of the three typical members (cytosolic, mitochondrial, and ER resident) of the HSP90 family (reviewed in [15]). The cytoplasmic member, more often dubbed HSP83 in* Leishmania*, is encoded by a large number of genes tandemly organized. Thus, for example, in the* L. major* genome there are 17 HSP90 (HSP83) genes tandemly organized (from gene LmjF33.0312 to gene LmjF33.0365). This high gene copy number is probably maintained because the high level of HSP90 protein that the parasites need to express. It was determined that, in unstressed* Leishmania* promastigotes, HSP90 make up 2.8% of the total protein [46]. In contrast, the ER paralogue of the HSP90 family, named Grp94, was found to be encoded by a single-copy gene [47]. Remarkably, this protein in* L. donovani* has been found to be involved in lipophosphoglycan (LPG) synthesis [48]; LPG is the predominant surface glycoconjugate in both procyclic and metacyclic stages. Finally, the mitochondrial member (named TRAP-1 or HSP75) of the HSP90 family is encoded in the* L. major* genome by gene LmjF33.2390, even though no further characterization of this protein has been described until now.

### 4.1. HSP90 as a Triggering Factor of Stage Differentiation in* Leishmania*

The ability of HSP90 machinery to sense and respond to environmental stimuli has been exploited by protozoan parasites such as* Plasmodium*,* Eimeria*,* Theileria*,* Toxoplasma* [49], and* Trypanosoma*, among others (reviewed in [50]). In* Leishmania*, the first evidence about the pivotal role played by HSP90 in stage differentiation from promastigote to amastigote was found in* L. donovani* by treatment of parasites with HSP90-specific drugs such as geldanamycin or radicicol. These chemotherapeutic agents bind to the ATP-binding pocket of HSP90, inhibiting specifically the function of the protein [40]. Treatment of* Leishmania* promastigotes with geldanamycin induces a morphological differentiation towards amastigote-like forms, and the expression of amastigote-specific proteins is concomitantly observed [51]. It should be noted that the morphological changes experienced by the parasites upon geldanamycin treatment were very similar to those induced by heat shock, which itself has also been shown to induce differentiation. A detailed biochemical characterization of the interaction between geldanamycin and the* L. braziliensis* HSP90 has shown that this compound inhibits the LbHSP90 ATPase activity with an IC_50_ of 0.7 *μ*M [52]. Thus, all these data suggest that a decrease in the levels of available HSP90 by either enrolment in the heat shock response or artificial inactivation by drugs may be the trigger for stage differentiation. In this regard, variations in the levels of* de novo* HSP90 synthesis were reported to exist naturally along the promastigote-to-amastigote differentiation process in axenic conditions [53]. Altogether, these findings point to the HSP90 cellular homeostasis as a key factor for the control of stage differentiation in* Leishmania* [6]. This idea is based also on the proved involvement of HSP90 in the regulation of cellular growth and differentiation in different organisms [38]. More recently, it has been suggested that HSP90 would be acting as a possible antagonist to HSP100, which has been linked to the amastigote-differentiation process [54].

### 4.2. HSP90 Cochaperones in* Leishmania*

As stated above, HSP90 is assisted by a plethora of cochaperones, which are involved in different stages of the HSP90 functional cycle, often contribute to the selection of client proteins chaperoned by HSP90, and facilitate client maturation by interacting with distinct regions on HSP90. So far, only limited information exists with regard to the complexes formed by HSP90 and its cochaperones in* Leishmania*. A comprehensive list of putative components of HSP90-heterocomplexes in several protozoan parasites, including* Leishmania* has been published recently [55]. The first experimentally confirmed cochaperone for HSP90 in* Leishmania* was STI1/HOP [56]. The synthesis of this protein is upregulated by heat shock, and coimmunoprecipitation experiments indicated that the* Leishmania* protein forms a salt-sensitive complex with HSP90 and HSP70. In* L. major*, the protein STI1 is encoded by gene LmjF08.1110. In recent works, indirect evidence on an essential role for STI1 in* L. donovani* promastigotes has been reported [57, 58]. STI1 is widely conserved and it has been found in diverse organisms; however, it is named differently depending on the organism. Thus, in yeast, the protein is also named STI1 whereas the mammalian orthologue is called HOP (HSP90/HSP70-organizing protein). The protein plays at least two relevant roles in the HSP90 functional cycle. First, it is an ATPase regulator (inhibitor) that binds to the EEVD sequence in the C-terminus and to other domains of HSP90 in the open conformation [59]. The second critical activity is the ability to simultaneously bind both HSP90 and HSP70 through distinct tetratricopeptide repeat (TPR) sites existing in the molecule [10]. Another gene, LmjF36.0070, is annotated in the* L. major* genome database as “stress-inducible protein STI1 homolog.” However, apart from the presence of TPR motifs, both proteins share low sequence conservation [15]. In a recent work, both alleles for the LmjF36.0070 orthologue, which was named “hop2,” were successfully deleted in* L. donovani* promastigotes; the resulting null line did not show growth defects or loss of infectivity for macrophages, suggesting a nonessential role for this protein at least for in vitro growth [60]. In the same work, gene encoding a protein similar to HIP (HSC70 interacting protein), another HSP90/HSP70 cochaperone, was found to be also dispensable for growth of* L. donovani* promastigotes and for in vitro multiplication in macrophages [60].

The orthologue to protein SGT (small glutamine-rich tetratricopeptide repeat), another cochaperone for HSP90/HSP70, has been also characterized in* L. donovani*. In this case, repeated failures in obtaining a null mutant line by gene-replacement point to an essential role of this protein for proliferation of the parasite [61]. SGT is encoded in the* L. major* genome by gene LmjF.30.2740. Coimmunoprecipitation analysis showed a direct interaction of SGT with HSP70, HSP90, and Sti-1/HOP [61].

The cochaperone Aha1 also plays a very relevant role in the ATP-driven HSP90 cycle [62]. The intrinsic ATPase activity of HSP90 is relatively weak, and Aha1 stimulates it several folds. The biochemical and biophysical characterization of* L. braziliensis* Aha1 (LbAha1) has been published recently [63]. LbAha1 was found to stimulate the ATPase activity of LbHSP90 by around 10-fold; in addition, a model in which two LbAha1 molecules interacts with the LbHSP90 dimer was postulated [63]. The gene encoding for Aha1 in the* L. major* genome database is annotated as LmjF.18.0210.

The cochaperone p23 associates with HSP90 late in the chaperone cycle (Figure 1), facilitating the maturation of client proteins [38]. In the* L. major* genome databases, two evolutionarily divergent p23-homologues, encoded by genes LmjF34.0210 and LmjF35.4470, were identified by sequence homology analysis [15]. In a recent work, both p23 proteins from* L. braziliensis* (named Lbp23A and Lbp23B) were produced as recombinant proteins in order to perform structural and functional studies [64]. Lbp23A and Lbp23B are the orthologs to the proteins encoded by genes LmjF35.4470 and LmjF34.0210, respectively. Both proteins were found to interact with* L. braziliensis* HSP90, inhibiting its ATPase activity [64]. Moreover, it was found that both proteins have intrinsic chaperone activity, based on its ability to prevent the thermal aggregation of two model proteins: malic dehydrogenase (MDH) and luciferase. Given the structural similarity existing between p23 and the *α*-crystallin domain of small heat shock proteins (sHSPs), it is possible that some of these* Leishmania* proteins might be actually a sHSP (see below).

Some cyclophilins (or immunophilins) have been found to be associated with HSP90 complexes and, therefore, categorized as cochaperones [65]. Cyclophilins are protein chaperones with PPIase activity, which catalyses the* cis-trans*-isomerization of peptidylprolyl bonds, affecting stability, activity, and localization of client proteins [66]. An in silico study has shown that the superfamily of immunophilins in* L. major* comprises 17 members [67]. Recently, the* L. donovani* chaperone cyclophilin 40 (*L. major* ortholog: LmjF.35.4770) has been shown to be essential for intracellular multiplication in macrophages [68].

To date, around 300* bona fide* HSP90 clients have been identified in different organisms. Among these, two dominant groups have been distinguished, specifically, transcription factors and kinases, most of which participate in signal transduction pathways of cell growth and differentiation. Other clients include DNA- and RNA-binding proteins (including polymerases), ribosomal proteins, small GTPases, cytoskeletal proteins, and ion channels. Also, many viruses hijack Hsp90s for maturation of their proteins [36]. However, in* Leishmania*, the number of putative client proteins of HSP90 that have been demonstrated experimentally to date is low. Adriano and coworkers [69] demonstrated the interaction between HSP90 and the cytoplasmic* Leishmania* silent information regulator 2 SIR2RP1. The SIR2RP1 ortholog in* T. brucei* has been described as a chromosome-associated NAD-dependent enzyme involved in DNA repair and catalyzes both deacetylation and ADP ribosylation of histones [70]. On the other hand, reverse genetic techniques have shown that SIR2RP1 is critical for survival and/or proliferation of* L. infantum* [71].

## 5. The HSP70 Family

Soon after discovering of heat shock proteins (HSPs) it became clear that the accumulation of a 70 kDa protein, which was designated heat shock protein 70 (HSP70), was intimately linked to the heat shock response and its accumulation was associated with an enhanced cell survival to stressful conditions [72]. Subsequent research revealed that HSP70 is essential for protection against stresses that cause protein denaturation, and that, in nonstressed cells, there exist constitutively expressed HSP70 proteins, which are performing house-keeping roles related with protein folding. Based on this finding these proteins were named also chaperones; the house-keeping functions of HSP70 chaperones include transport of proteins between cellular compartments, removal of misfolded proteins, folding and refolding of proteins, prevention and dissolution of protein aggregation, and control of regulatory proteins. HSP70s also cooperate with other ATP-dependent chaperones including HSP90 and chaperonins to fold some substrates, and with certain proteins of the AAA+ family to dissociate aggregates of misfolded proteins (reviewed in [73–75]).

As a reflection of its essential and crucial functions, HSP70 is, by far, the most conserved protein present in all organisms [76]. Moreover, most eukaryotic organisms have several HSP70 variants localized in all major subcompartments, the cytosol, the nucleus, ER, mitochondria, and also chloroplasts [74, 77]. For example, the yeast* Saccharomyces cerevisiae* contains nine Hsp70 homologs [77]. Similarly, the human HSP70 family comprises ten members that differ from each other by amino acid sequence, expression level and subcellular localization [77]. Even in* Escherichia coli* (K-12), three distinct HSP70 (DnaK) genes exist [78]. Among parasites, six structural different HSP70s are present in* Plasmodium falciparum* [79] and nine different members of the HSP70 family are encoded in the genome of* Leishmania* (Table 2) and related trypanosomatids [15]. HSP70 family members located in the endoplasmic reticulum are known as BiP or Grp78, and those found in the mitochondria are named mtHsp70, Grp75, or mortalin (a term only used when referring to mitochondrial HSP70 members in mammals).

The eukaryotic HSP70s constitute a super-family consisting of two major families, recently classified as the typical HSP70 family (described above) and the atypical HSP110/GRP170 family [80]. The domain architectures of HSP110 and Grp170 are very similar to that of HSP70s; the main differences lie in large acidic insertions between the peptide binding domain (PBD) and the lid, and also at the C-terminus. Whereas the function of the Grp170 group is limited to ER, HSP110s are found in the cytoplasm. Although their biochemical roles remained elusive, it is belief that they are not active as protein folding chaperones. Mammalian Hsp110 and yeast Sse1p were shown to functionally and physically interact with prototypical HSP70s, acting as potent nucleotide exchange factors (NEFs) for the HSP70 counterpart [81].

### 5.1. The HSP70 Chaperone Machine

All cellular functions of HSP70 chaperones seem to depend on an ATP-driven mechanism by which polypeptides are bound to and released from HSP70 [82]. It has been suggested that the ATP-driven cycle of HSP70 operates by a bidirectional heterotrophic allosteric mechanism [83]. The structure of the different members of the Hsp70 family is highly conserved, consisting of an N-terminal ATPase domain (also named adenine nucleotide-binding domain or NBD) of c. 45 kDa and a C-terminal substrate binding domain of c. 25 kDa, which is further subdivided into a beta-sandwich subdomain with a peptide-binding cleft and a C-terminal alpha-helical subdomain that acts as a lid, covering the peptide binding site [73]. The interdomain linker, connecting the NBD to the peptide binding domain (PBD), is highly conserved and plays a critical role in the allosteric regulation during the HSP70 functional cycle [84]. Also, many HSP70s also contain a G/P-rich C-terminal region that ends in the EEVD-motif, which mediates their binding to the TPR-domain containing cochaperones. The TPR is characterized by a 34-amino acid motif that forms an antiparallel *α*-helical hairpin [85]. Two important cochaperones, HOP and CHIP, bind EEVD-motif (also present in the C-terminal end of HSP90) by their TPR domains. In fact, the TPR-protein HOP forms a link between HSP90 and HSP70 (Figure 1), facilitating protein client transfer between both chaperones [86].

**Figure 2:**
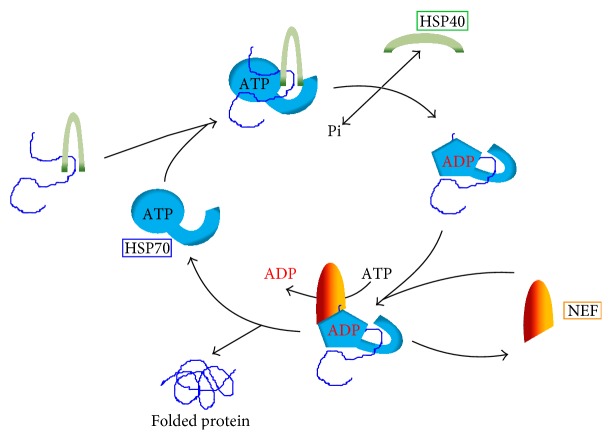
Overview of the HSP70 chaperone cycle. In the initial step, the substrate (unfolded protein), bound to the HSP40, forms a complex with the HSP70 in its ATP-loaded state. ATP hydrolysis promotes the transfer of the substrate to the peptide-binding pocket of HSP70. Following substrate transfer, HSP40 leaves the complex and the nucleotide exchange factor (NEF) is recruited to the HSP70-substrate complex, stimulating the ADP-ATP exchange in HSP70. ATP binding to HSP70 induces a conformational change leading to the release of both NEF and substrate.

Figure 2 shows the so-called ATPase cycle of HSP70, consisting of an alternation between the ATP-bound state, with low affinity and fast exchange rates for polypeptide substrates, and the ADP-bound state, with high affinity and low exchange rates for substrates [87]. Although ATP hydrolysis is basic for chaperone activity of HSP70s, the intrinsic ATP hydrolysis rates of HSP70s are generally low. Nevertheless, this low intrinsic ATPase activity is stimulated by the recruitment and binding of J-type chaperones of the HSP40 family (see below) and the polypeptidic substrate [88]. Thus, the encounter between the substrate and the HSP70-ATP complex results in ATP hydrolysis, and, as a result, the substrate is trapped in the HSP70-ADP complex, which has a low peptide dissociation rate. The modulation of the affinity for polypeptidic substrates would be triggered by a conformational change in the lid domain that, upon ATP hydrolysis, closes on the substrate that is located within the PBD. This high affinity of the ADP-bound state of HSP70s to unfolded polypeptides allows these chaperones to prevent efficiently the aggregation of misfolded proteins. Also, it has been proposed that HSP70 uses the energy of ATP hydrolysis to recruit a force of entropic origin to locally unfold aggregates or pull proteins across membranes [87, 89].

HSP70s are assisted by a group of cochaperones, being the most important the family of HSP40 (also known as DnaJ-like proteins or J domain proteins, JDPs) and several molecules acting as nucleotide exchange factors (NEFs). JDPs, in the present of the polypeptidic substrate, stimulate greatly the ATPase activity of HSP70s [90]. HSP40 is thought to act as the primary substrate recruiter for HSP70 and to stimulate the HSP70 ATPase activity. In most organisms, the HSP40 family is composed by many members, and* Leishmania* is not an exception (see below).

In order to release the polypeptide from the hsp70-substrate, taking into account the high affinity of HSP70 to the substrate in the ADP-bound state and the physiological ATP concentrations, the engagement of NEFs to the HSP70-substrate complexes is crucial. Thus, NEFs trigger the dissociation of bound ADP from HSP70 to allow the binding of ATP, resetting the cycle (Figure 2). To date, four different families of NEFs for Hsp70s have been described: the GrpE family in prokaryotes and organelles of prokaryotic origin, the Bag family in the eukaryotic cytosol, the HspBP family in the eukaryotic cytosol and endoplasmic reticulum, and HSP70-related proteins in the eukaryotic cytosol (HSP110) and endoplasmic reticulum (Hsp170 or Grp170) [20, 91]. These four groups of proteins have entirely different structures; therefore, their nucleotide exchange role seems to be arisen by convergent functional evolution.

Some other proteins have also been identified as HSP70 cofactors, such as HIP, HOP, and CHIP [92]. HIP interacts with the ATP-binding domain of the HSP70, and it cooperates with J domain proteins in increasing ATP hydrolysis and further stabilizes the HSP70-substrate interaction [93]. HOP binds to the carboxyl-terminal domain of HSP70, and also to HSP90, mediating the association of these molecular chaperones [86]. Human CHIP is a cytosolic protein containing three TPR domains at its amino-terminal moiety, by which this cochaperone interacts with the carboxyl-termini of both HSP70 and HSP90. In addition, CHIP possesses a U-box domain endowed with E3 ubiquitin ligase activity. Hence, CHIP provides a link between the processes of protein folding and protein degradation [94].

Some structural features of HIP from* L. braziliensis* have been studied in a recent work, and the results indicated that the protein forms a dimer with an elongated structure, similar to the HIP protein in mammalians [95].

### 5.2. Chaperones of the HSP70 Family in* Leishmania*

**Figure 3:**
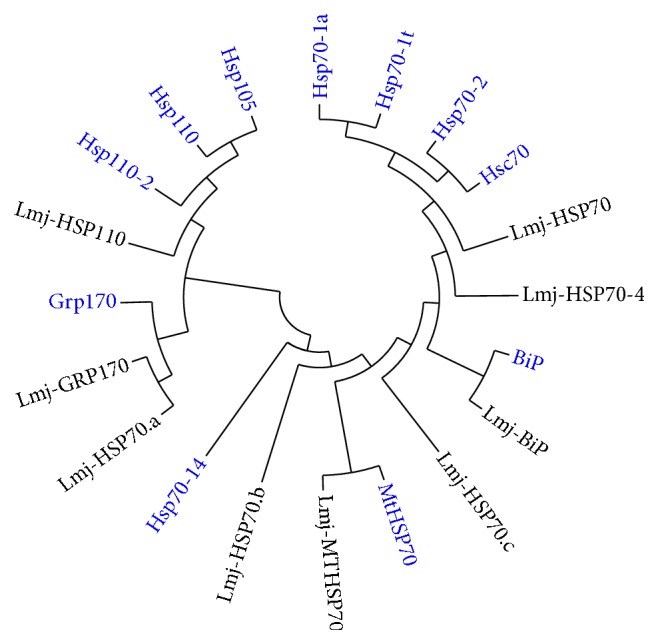
Relationships inferred from the amino acid sequence similarities existing among the members of human and* L. major* HSP70 families. The phylogeny was inferred using the neighbor-joining method. The optimal tree with the sum of branch length = 7.43402253 is shown. The evolutionary distances were computed using the Poisson correction method. All positions containing gaps and missing data were eliminated. There were a total of 474 positions in the final dataset. Evolutionary analyses were conducted in MEGA6 [8]. See Table 2 for further details of the protein sequences used for this analysis.

In Table 2 are listed the members of the HSP70 encoded in the* L. major* genome and their putative orthologues existing in humans. The phylogenetic tree shown in Figure 3 was constructed with the aim of illustrating possible relationships existing between the different members of* Leishmania* HSP70 family and the closest orthologs in humans. Thus, this analysis confirmed the existence in* Leishmania* of all HSP70 groups composing the eukaryotic HSP70 family: cytosolic HSP70s, ER HSP70 (BiP), mitochondrial HSP70, and HSP110 subfamily. This study shows that* Leishmania* contains two distinct members of the Grp170 group, that is, GRP170 and HSP70.a, whereas a sole member exists in humans. Additionally,* Leishmania* parasites possess three HSP70 variants (HSP70.4, HSP70.b, and HSP70.c) that have no clear relatives in humans. Conversely, orthologue to the human Hsp70-14 does not seem to exist in* Leishmania*. Among the atypical HSP70s in* Leishmania*, the localization of HSP70.4 in* L. major* was investigated by indirect immunofluorescence and the results indicated that the protein is cytoplasmic, even in periods of stress [96]. Interestingly, the phylogenetic analysis (Figure 3) shows that HSP70.4 groups with prototypical cytoplasmic HSP70s. Sequence analysis of* Leishmania* HSP70.b indicates that the protein is more similar to cyanobacterial DnaK than to the mitochondrial HSP70s [15]. As cyanobacteria are considered the origin of chloroplasts [97], this finding may represent a further support to the idea that the ancestor of* Leishmania*, and the rest of trypanosomatids, possessed chloroplasts [98, 99]. Nevertheless, current location of HSP70.b in* Leishmania* remains to be determined. An indirect indication of the relevance of the different members of HSP70 superfamily is found in the fact that an equivalent complement of HSP70s exists in two other trypanosomatids:* T. brucei* and* T. cruzi* [15, 100], which contains orthologues for all the subdivisions of the* Leishmania* Hsp70 protein family. Recently, the HSP70.c ortholog in* T. brucei* has been biochemically characterized regarding its chaperone function [101]. It was shown that this protein, located in the cytoplasm, interacts with Tbj2 (a member of HSP40 family; ortholog to protein J2; see Table 3). Both proteins, expressed and purified as recombinant proteins, are active in preventing thermal aggregation of MDH and rhodanese. Moreover, it was found that Tbj2 stimulates the ATPase activity of TbHsp70.c [101].

Genes encoding for prototypical HSP70s were among the first genes to be cloned in* Leishmania*, because of their presumed importance in its life cycle, and also by technical reasons; that is, the high evolutionary conservation of HSP70 genes allowed their identification by hybridization using heterologous genes as probes (reviewed in [15]). In the* Leishmania* species analyzed to date, the HSP70 locus consists of six-seven gene copies arranged tandemly in a head-to-tail organization [102, 103]. The first five genes (named HSP70-I) are identical, whereas gene 6 (HSP70-II), located at the 3′-end of the locus, differs only in the sequence of the 3′-untranslated region (UTR). These 3′-UTRs regulate the translational efficiency of the HSP70 mRNAs. Thus, while the HSP70-I transcripts are associated with ribosomes at both normal and heat-shock temperatures, HSP70-II transcripts are translated specifically during heat shock [104]. Although promastigotes lacking the HSP70-II gene could be obtained, they showed many cellular and biochemical alterations, together with a reduced virulence [105].

Upon being taken up by macrophages, parasites are exposed not only to heat shock, but also to various macrophage defense mechanisms including release of reactive oxygen species, all of which heighten parasite oxidative stress. Experimentally, it has been demonstrated that a previous heat shock treatment of* Leishmania* (mimicking natural heat shock occurring during transmission to the vertebrate host) leads to increased parasite resistance to oxidative stress. A direct involvement of HSP70 in resistance to macrophage induced oxidative stress was determined with experiments in which HSP70 was overexpressed in* L. chagasi* promastigotes [106].

## 6. Chaperonins

Chaperonins are divided into two groups: group I chaperonins (or HSP60) are mostly found in prokaryotes, mitochondria, and plastids, and group II chaperonins (TRiC/CCT) are in archaea and the eukaryotic cytosol [107, 108]. The best characterized chaperonin of group I is the GroEL protein of* E. coli* [109]. The GroE machinery consists of 14 GroEL subunits, arranged in a cylinder of two heptameric rings, to which the cochaperone GroES, also a heptameric ring, binds. Inside each ring, a large cavity with a lining of largely hydrophobic amino acid residues allows interacting with and accommodating unfolded polypeptide chains up to a size of about 50 kDa. Remarkably, each one of the two rings, alternately, experiences cycles of ATP binding and hydrolysis. Upon ATP binding, the GroEL ring recruits the cochaperonin GroES ring, which will cap the cavity; the assembly of the GroES ring induces a dramatic structural reorganization in the GroEL subunits and the enclosed chamber changes from hydrophobic lining (in the open ring) to a hydrophilic lining [110]. This decrease in hydrophobicity would favor the folding of nonnative polypeptides with exposed hydrophobic surfaces that were trapped inside the open GroEL ring [111]. Once the substrate is encapsulated in the chamber, the slow hydrolysis of ATP (half-time ~ 10 s) provides time for correct folding of the encapsulated polypeptide. ATP hydrolysis is not required for protein folding; rather, ATP hydrolysis in the GroES-bound ring is a prerequisite for ATP binding to the opposite ring. Currently, the bacterial chaperonin GroE machinery is the best understood among the chaperone systems devoted to folding of nascent or stress-denatured polypeptides [11]. The closely related proteins to GroEL and GroES in mitochondria are called HSP60 and HSP10, respectively.

The architecture of the CCT (or TRiC) machinery resembles that of GroE machinery; group II chaperonins also form large cylindrical oligomers consisting of two rings arranged back to back. Each ring contains a central cavity in which nonnative proteins are encapsulated in an ATP-dependent process for folding into the native state [112]. In group II, an extra protein domain replaces group I cochaperonin GroES/HSP10 [113].

Another major difference between the two groups of chaperonins is that those of group I form homomultimers, while there is a heterooligomeric assembly in group II. In yeast, for example, the CCT or TRiC machinery is a large cylindrical 900-kDa oligomer composed of a double-ring structure. Each heteromeric ring contains eight different 60-kDa subunits surrounding the cavity where substrates are folded [114]. All eight subunits, Cct1 to Cct8, are essential and expressed constitutively under normal conditions. The subunits share 30 to 35% of sequence identity, with the highest level of conservation within the ATPase domain, and the substrate-binding domains being highly divergent [114].

An* L. major* HSP60 gene was cloned, and the protein was shown to be a prominent antigen recognized by sera from leishmaniasis patients [115]. In* L. donovani*, two distinct HSP60 genes were identified [116]. One of the genes,* cpn60.1*, though actively transcribed, is not expressed to detectable levels of protein in axenically cultured parasites. In contrast, the other gene,* cpn60.2*, is really expressed as protein and its abundance increases 2.5-fold under heat shock and in axenic amastigotes (in comparison with the levels present in promastigotes). As expected, this HSP60 locates in the mitochondrial matrix [116]. In* L. major*, the orthologs are LmjF.32.1850 (*cpn60.1*) and LmjF.36.2020 (*cpn60.2*). Two additional HSP60 coding genes (LmjF30.2820 and LmjF36.2030) were identified after mining the* L. major* genome database [15]. The existence of several HSP60 isoforms has been observed in some bacteria and also in eukaryotes. Thus, for example,* Mycobacterium tuberculosis* contains two chaperonins, termed 60.1 and 60.2, that exhibit around 61% sequence identity. Attempts to inactivate these genes reveal that only the gene encoding the 60.1 protein can be inactivated [117]. Remarkably, the chaperonin 60.1 protein of* M. tuberculosis* turned out not to be a molecular chaperone (it does not fold proteins) but to be a potent virulence factor. Another example,* Drosophila melanogaster* has four Hsp60 proteins and each seems to serve a distinct function [118]. Thus, it is likely that some HSP60 variants are playing functions other than protein folding in the mitochondria.

Regarding cochaperonin HSP10, it has been studied to date only in* L. donovani*, where it is expressed preferentially under heat shock conditions. Accordingly, the abundance of HSP10 increased significantly during in vitro differentiation to the amastigote stage. Furthermore, it was shown that the protein localizes in the mitochondrion and coprecipitates with HSP60 [119]. In* L. major*, HSP10 is encoded by two genes (LmjF26.0620 and LmjF26.0640) with almost identical sequence [15].

## 7. The HSP40/DnaJ Superfamily

HSP40s, also known as dnaJ-like or J domain proteins, are crucial partners for HSP70 chaperones, and much of the functional diversity of the HSP70s is driven by this diverse class of cofactors [16]. The prototypical and founding member of this superfamily is the* E. coli* DnaJ protein [120]. In general, HSP40s regulate the HSP70 cycle through stimulation of ATPase activity of HSP70. ATP hydrolysis is essential for stabilization of the interaction between HSP70 and its substrates (Figure 2).

Remarkably, HSP40s, in a cell or in a cellular compartment, outnumber HSP70 variants. Thus, six DnaJ homologues have been found in* E. coli*, 41 DnaJ/HSP40 family members in humans [121], and 43 DnaJ-related proteins in* P. falciparum* [122, 123]. Furthermore, J proteins show a large degree of sequence and structural divergence, in agreement with the idea that they greatly contribute to the multifunctionality of the HSP70 machinery [16]. The presence of the J domain is the family mark for these proteins, and it is essential for their functional interaction with the HSP70 partner. The J domain is comprised of approximately 70 amino acids and it exhibits four alpha-helices (I–IV); the existence of a highly conserved His-Pro-Asp (HPD) tripeptide motif in the loop region between helices II and III is another structural feature of the J domain [122]. The HPD tripeptide seems to be essential for HSP40 functionality, since variations within the HPD motif are known to abolish the stimulation of HSP70 ATPase activity by HSP40 [124]. In addition to the J domain, there are two regions in the prototypical DnaJ: a Gly/Phe-rich region (GF domain) and a cysteine-rich zinc-binding domain [125]. The zinc-binding domain is characterized by the presence of four cysteine-repeat sequences (CXXCXGXG), capable of coordinating the binding of two zinc ions.

The classification of this large and diverse group of HSP40 and HSP40-like proteins is a challenge, although the existence of three categories of HSP40s is widely accepted [125–127]. Type I HSP40s contain, similar to the* E. coli* DnaJ, the three domains: the J domain, a Gly/Phe-rich region, and a zinc-finger domain. Type II HSP40s have the J domain and the Gly/Phe-rich region but lack the cysteine-rich motif. Finally, type III HSP40s do not have any of the DnaJ-typical motifs other than the J domain. More recently, Louw and coworkers have proposed a fourth group (type IV) that is composed of HSP40s having a J-like domain in which the highly conserved HPD sequence motif is corrupted [100]. Aside from the above conserved domains or regions, some DnaJ family members contain additional domains, which may determine the functional diversity of DnaJ proteins. Also, more recently, the presence in some DnaJ proteins of a dimerization domain at its C-terminal region has been found to be essential for their chaperone activity ([121] and references therein).

A search in the genome of trypanosomatids for proteins containing the J domain in their sequence showed the existence of an enormous number of HSP40s in these parasites: 67 in* T. cruzi*, 66 in* L. major*, and 65 in* T. brucei* [15]. For this review, we have inspected again the* L. major* annotated genome and three new members were discovered. Thus, at least, 69 putative HSP40s (J proteins) were found to be encoded in the* L. major* genome (Table 3). Also, following the structural rules indicated above, the proteins have been grouped within types I–IV. Hence, eight* Leishmania* HSP40s may be considered as J proteins type I, since they contain all three domains of prototypical DnaJ protein. Another 19 HSP40s belong to the type II category as they have Gly/Phe-rich segments close to the J domain but lack an apparent Zn-binding domain. The largest category is that formed by proteins containing only the J domain, 37 of them are type III and the other 4 are type IV. Among the putative HSP40s listed in Table 3, there is one that lacks the J domain (protein J30); nevertheless, it was included in the list because its ortholog in* T. brucei* (Tb927.8.1010) contains the J domain at the N-terminal moiety. Thus, the* L. major* J30 seems to be a truncated version, which lacks the N-terminal region (220 amino acids) of* T. brucei* J30 protein. Although the J domain is located at the N-terminal region in most* Leishmania* HSP40s, there are some J proteins in which the domain is located at the C-terminal region and a few of them in which the J domain is located in the middle of the molecule. This atypical location of J domain in some HSP40s has been also described for J proteins in yeast [126]. Noticeably, one of the* Leishmania* HSP40s, J14, contains two J domains. Another remarkable finding is the existence of TPR domains in several J proteins; thus, J50, J52, J53, and J67 contain two TPR domains each, whereas a sole TPR domain is present in J42 and J65 (Table 3). As stated above, the TPR domain has been found to be a docking site, interacting with the EEVD motif present at the C-termini of some HSP70 family members and in the C-terminus of HSP90.

In spite of this overwhelming number of HSP40s existing in* Leishmania*, none of these molecules have been biochemically characterized to date, and consequently nothing is known about those potential HSP70-HSP40 partnerships. However, indications about the relevance in the* Leishmania* biology are being glimpsed for some of them based on proteomic studies addressing posttranslational modifications occurring during the differentiation process. Thus,* L. infantum* J2 protein has been found to be phosphorylated on Serine-89, and the phosphorylation of this residue increased by 3.5-fold at 2.5 h of promastigote-to-amastigote differentiation, reaching an increase in phosphorylation of 22-fold in full differentiated amastigotes [128]. Such a dramatic increase in phosphorylation suggests a relevant role for this protein in the differentiation process from promastigote to amastigote stage.

## 8. Small Heat Shock Proteins (sHSPs)

They comprise the most poorly conserved family of molecular chaperones, showing high heterogeneity both in sequence and size [129]. Their common trait is the conserved *α*-crystallin domain (ACD; PROSITE profile PS01031), which was firstly described in the eye-lens protein *α*-crystallin, hence, its name. The ACD is formed by seven or eight antiparallel *β*-strands that form a *β*-sandwich; however, the domain shows low sequence identity among the different sHSPs. Thus, unlike other families of HSPs, such as the HSP70 and HSP90 chaperone families, sHSPs show extensive sequence variation and evolutionary divergence [18].

The *α*-crystallin domain is responsible for dimer formation, which represent the basic structural unit of many sHSPs [130]. Additionally, their amino- and carboxyterminal extensions are involved in modulating oligomerization, substrate binding and chaperoning function [131]. sHSPs can form large dynamic oligomers and coaggregate with aggregation-prone proteins for subsequent, efficient disaggregation. They act in an ATP-independent fashion, but the release of substrate proteins from the transient sHSP reservoirs and their refolding often require cooperation with ATP-dependent chaperone systems, mainly the HSP70 machinery [18]. In essence, the functional role of sHSPs would be avoiding aggregation of misfolded and/or unstable proteins even in the absence of stress. Nevertheless, it is unclear how sHSPs recognize nonnative substrates and to what extent they may show substrate specificity.

sHSPs bind a wide range of cellular substrates; they are implicated in many different cellular functions and also in cellular defenses against different stresses, such as high temperature and oxidative stress [132]. On the other hand, sHSPs have been shown to be associated with membranes, although they do not contain transmembrane domains or signal sequences. Via specific membrane lipid interactions, sHSPs have been shown to modulate major attributes of the membrane lipid phase such as the fluidity, permeability, or nonbilayer propensity [133]. Furthermore, recent studies have shown that some sHSPs localize to the mammalian and plant ER, in which sHSPs would play protective effects, being indeed relevant in maintaining ER homeostasis [134].

For many organisms, different sHSPs have been described, but they are the result of independent evolution processes that have taken place in major groups of organisms. Thus, many orthologs of all 10 human sHSPs have been identified in other mammals, but only distinct subsets are found in other vertebrates, and different vertebrates have unique paralogs [18]. In* Leishmania*, and related trypanosomatids, the number of sHSPs seems to be low. In fact, until recently, a sole sHSP (HSP20,* L. major* ID: LmjF.29.2450) was identified in this group of parasites [15]. However, apart from a report on the immunoprophylactic potential of* L. amazonensis* HSP20 [135], studies about its functional relevance have not been conducted yet.

In a recent work, using human p23 cochaperone sequence and the BLAST algorithm, two additional ACD-containing proteins have been identified in* L. donovani* [136]. They have been named HSP23 (the ID for the* L. major* ortholog is LmjF.34.0210) and p23 (*L. major* ID: LmjF.35.4470). In a previous work [15], using a similar BLAST search, both proteins were identified as putative p23 (HSP90 cochaperones; see above). The reason is that both classes of proteins, sHSPs and p23, adopt topologically similar but sequentially unrelated structures [137]. The functional relevance of the atypical HSP23 has been studied* L. donovani* [136]. The protein is a stress-inducible protein with a threefold higher abundance in early amastigotes. Furthermore, generation of HSP23-null mutants allowed concluding that HSP23 is essential for* Leishmania* stress tolerance. In fact, a HSP23 null mutant line of* L. donovani* was found to be completely unable to survive at mammalian tissue temperature and, consequently, these HSP23-null mutants were noninfectious to primary macrophages in vitro [136]. Furthermore, in this study, it was shown evidence that HSP23 also protects against trivalent antimony (Sb^3+^), the active principle of pentavalent antimony, one of the main drugs used in clinic against leishmaniasis.

## 9. Chaperones in the Endoplasmic Reticulum and the Unfolded Protein Response (UPR)

The endoplasmic reticulum (ER) is the biggest organelle in most cell types and it is the point responsible for protein synthesis and maturation destined for the secretory pathway. In eukaryotic cells, almost all secreted proteins enter the ER either during (cotranslational) or soon after their synthesis. After translocation, proteins are folded by the chaperone machinery in the ER. Additionally, many proteins that enter the ER will be posttranslational modified by glycosylation. The lectins calnexin (Cne1 in yeast) and calreticulin are key components in the quality control of glycoprotein folding [138, 139]. Many secreted proteins contain disulfide bonds that maintain their tertiary or quaternary structures; however, when disulfide bonds are formed incorrectly or fail to form, protein misfolding occurs. In order to resolve the improper covalent disulfide links, protein disulfide isomerases (PDIs) catalyze the formation, reduction, and isomerization of disulfide bonds. Therefore, PDI may be considered as* bona fide* molecular chaperones.

On the other hand, a retrograde transport of aberrant polypeptides from the ER into the cytosol for proteasomal degradation also exists. This pathway is known as “ER-associated degradation” (ERAD). When unfolded proteins accumulate in the ER, the UPR pathway is activated.

Very little is known about the ER secretory pathway in* Leishmania* regarding its function in protein folding, quality control, and stress response. This gap in our knowledge is somewhat lower after the recent publication of a review about ER stress responses in* Leishmania* [140]. For the purpose of this review, we outline the ER chaperones identified in* Leishmania* and the roles played in this organelle regarding protein folding and secretion.

As shown above, the ER members of the families HSP70 (BiP) and HSP90 (GRP94) have been identified in* Leishmania*. In this regard, a direct interaction between* L. donovani* BiP and protein A2 has been documented [141]. The A2 protein is expressed predominantly in* L. donovani* amastigotes, but it is absent in* L. major* (the* A2* ortholog is a pseudogene in this species). It has been suggested that A2 is a virulence factor responsible for viscerotropic survival of* L. donovani* in the mammalian host. On the other hand, accumulation of BiP in the ER was observed after treatment of* L. major* promastigotes with the protein glycosylation inhibitor tunicamycin. Thus, the upregulation of the chaperone BiP, as part of the UPR response, would increase the protein folding activity and prevent protein aggregation, alleviating the ER stress induced by impaired glycosylation [142].

Another ER molecular chaperone studied in* Leishmania* is calreticulin (*L. major* gene: LmjF.31.2600). Overexpression of a truncated form of calreticulin in* L. donovani* leads to decreased secretion of acid phosphatases (one of the major secreted glycoproteins), and a lower survival rate of the parasite into the macrophages. The authors of this study suggest that altering the function of an ER chaperone such as calreticulin in* Leishmania* affect the trafficking through the secretory pathway of proteins that are associated with the virulence of the parasite [143]. Similarly, overexpression of the mutated/inactive form of an* L. donovani* protein disulfide isomerase (PDI) led to a reduction in the release of secretory acid phosphatases, suggesting that PDIs also play a critical role in the* Leishmania* secretory pathway [144]. The ortholog in* L. major* for this PDI is coded by gene LmjF.06.1050.

## 10. Molecular Chaperones of the Mitochondrion

The vast majority of mitochondrial proteins are encoded in the nucleus and synthesized in the cytosol. Therefore, many proteins must be posttranslationally translocated in an unfolded state into the mitochondria. The relevance of the process is evidenced when considering, for example, that mitochondria in humans retain only 13 protein coding genes, but the total number of different mitochondrial reaches over a thousand. Moreover, this protein traffic represent a unique challenge, given the presence of two distinct membrane systems, an inner membrane (IM) and an outer membrane (OM) in this organelle. To deal with this task, mitochondria possess dedicated chaperone systems to assist in these processes. Thus, mitochondria contain transport machineries in both the IM and the OM for the import of nuclear-encoded proteins. Components of the OM are named Tom proteins and the components of the IM are named Tim proteins. The structure of molecular complexes has been studied mainly in the mitochondria of the yeast* S. cerevisiae* and the fungus* Neurospora crassa* (reviewed in [145]). The proteins to be translocated into the mitochondria, presumably transported by cytoplasmic chaperones, are partially unfolded and interact through their exposed hydrophobic regions with Tom20, Tom22, or Tom70 proteins. Roughly, mitochondrial preproteins are thus targeted to mitochondria either via amino-terminal presequences to Tom20/Tom22 or via internal targeting sequences to Tom70. Subsequently, proteins are transferred to the general import pore (GIP), a multiprotein complex formed by at least five different proteins. The preproteins pass through the GIP and are inserted into the IM import channel, which is formed by a complex of proteins containing Tim23 and the mitochondrial member of the HSP70 family. Hence, a fraction of the mtHSP70 is bound to the TIM23 complex and serves as the ATP-dependent motor protein for mitochondrial protein import. The difference of potential between the mitochondrial intermembrane and matrix also contributes to protein import of preproteins. During import, cycles of ATP-hydrolysis by mtHSP70 generates the pulling force to introduce the preprotein into the mitochondrial matrix; in the process, the nucleotide exchange factor, Mge1, and two J domain proteins (Pam16 and Pam18) are also involved. Since preproteins cross the membrane in a completely unfolded conformation, due to the small pore diameter of the translocase complexes, mtHSP70 is also the first chaperone that initiates the folding process of the imported polypeptide [146]. In agreement with its evolutionary origin, mtHSP70 is more closely related to the bacterial DnaK than to its eukaryotic cytosolic counterparts [147].

Apart from the role of mtHSP70 in the cytoplasmic-mitochondrial transport, it also participates in the folding of those proteins that are synthesized by the mitochondrial translation apparatus and it protects the mitochondrial proteins from misfolding caused by chemical modifications, as those produced by reactive oxygen species (ROS), and other stresses. During folding reactions in the matrix, mtHSP70 cooperates closely with the other main chaperone type in the matrix compartment, HSP60 (see section chaperonins). A distinction between the functions in protein translocation and in protein folding is mainly achieved by the particular J-family cochaperone that interacts with mtHSP70 during these processes. The protein Mdj1 (the “mitochondrial DnaJ” homolog) is involved in mtHSP70-mediated folding reactions in the matrix compartment [146].

The existence of two distinct mtHSP70s in* Leishmania* is noticeable as usually a sole protein is present in the mitochondria of most organisms, even in related trypanosomatids [148]. At the amino acid level, the two proteins share an overall identity of 91% with the N-terminal end being completely conserved. Interestingly, expression analysis showed that the two* Leishmania* mtHSP70s are differentially expressed, one is predominant in promastigotes and the other is restricted to the amastigote stage [148]. In a recent work, the mtHSP70 machinery (mtHSP70, mtHSP40, and Mge1) has been studied in the* Leishmania*-related trypanosomatid* T. brucei* [149]. Based on the information provided in this study, it was possible to identity the* L. major* homologues for mtHSP40 and Mge1 as the genes LmjF35.2980 (J50; Table 3) and LmjF30.0730, respectively. Remarkably, in the work by Týč et al. [149], using RNA interference- (RNAi-) mediated depletion of each of these three proteins, evidence was obtained that the entire mtHSP70 machinery plays a relevant role in mitochondrial DNA replication and maintenance.

In an outstanding work, Teixeira and coworkers [150] have elegantly shown that mitochondrial peroxiredoxin mTXNPx in* L. infantum* functions as a crucial chaperone, allowing* L. infantum* to deal successfully with protein unfolding conditions during the transition from insect to the mammalian hosts. Peroxiredoxins (Prxs, EC 1.11.1.15) are ubiquitous antioxidant enzymes, found in all kingdoms of life, that help to control intracellular peroxide levels [151]. Recent studies suggested that Prxs may eject also molecular chaperone functions; however, little is known about the precise mechanism of chaperone function and its physiological significance. The studies with* L. infantum* mTXNPX have demonstrated that, in this parasite, the reduced form of the protein is a stress-specific chaperone reservoir, which is activated rapidly upon exposure to unfolding stress conditions [150]. In a previous article, the group showed that mTXNPx-deficient promastigotes are significantly more sensitive to a temperature shift from 25°C to 37°C than wild-type promastigotes and, consequently, the mTXNPx-deficient parasites were unable to survive within the mammalian host [152]. Interestingly, these phenotypes could not be attributed to the peroxidase function of mTXNPx, because an* L. infantum* line expressing a peroxidase-inactive variant of mTXNPx was fully capable of surviving the temperature shift to 37°C and infecting mice. These results suggest that the essential function observed in vivo is not based on the peroxidase activity but more likely involves a second function of mTXNPx, presumably a chaperone role. This atypical chaperone function has been demonstrated recently [150]. The reduced mTXNPx, structured as a decameric complex interacts with unfolded proteins, protecting them against temperature-induced aggregation. Therefore, mTXNPx is a relevant player of the mitochondrial proteostasis network, and its role as chaperone is crucial for parasite infectivity.

## 11. Selective Targeting of Chaperones as a Therapeutic Strategy against Leishmaniasis

HSP90, HSP70 and their cochaperones have been shown to be critical for the growth of a variety of different human tumor cell lines in which these chaperones are expressed at elevated levels. Therefore, these HSPs are currently considered as potentially targets in cancer and the inhibitory activity of many compounds are being experimentally addressed [153].

To date, there are few studies addressing the effect of molecular chaperone inhibitors as leishmanicidal drugs. In recent studies, the in vitro effect of 17-(allylamino)-17-demethoxygeldanamycin (17-AAG) against* L. amazonensis*,* L. infantum*,* L. major*, and* L. panamensis* promastigotes was demonstrated [154, 155]. Moreover, the in vitro treatment of* Leishmania*-infected macrophages with nanomolar concentrations of 17-AAG promoted the clearance of parasite infection [154]. The compound 17-AAG is a potent inhibitor for HSP90, and it is currently in clinical trials for cancer treatment [156]. Additionally, in preclinical studies with mouse models, 17-AAG has been found to inhibit the growth of* P. falciparum* and* Trypanosoma evansi* [157]. In a recent report, it has been demonstrated that 17-AAG is also efficient in reducing* L. braziliensis* promastigote growth, macrophage infection, and, more importantly, the parasite multiplication in vivo (BALB/c mice), highlighting its potential as a novel chemotherapy agent against cutaneous leishmaniasis caused by* L. braziliensis* [158].

## 12. Chaperones and Drug Resistance in* Leishmania*: The Other Side of the Coin

Control of leishmaniasis relies mostly on chemotherapy [3], given the lack of an effective vaccine and the difficulties to control the vector (sandfly). Nevertheless, treatment failure is increasing due to the emergence of parasites resistant to the most common antileishmanial drugs in several parts of the world, and most notably in India [159].

Pentavalent antimony, Sb(V), is the first line drug for patients with leishmaniasis in most parts of the world but acquired resistance against antimonials is high and common in some parts, such as the Indian subcontinent. Given the relevance of stress proteins in protecting cells from toxic external stimuli, it is not surprising the association observed between drug resistance and increased levels of these proteins in many types of cells, and this is true also for* Leishmania*. Thus, antimony resistant* L. tarentolae* promastigotes accumulate 4-fold more HSP70 than the wild-type cells [160]. Moreover, when the HSP70 was overexpressed in this* Leishmania* species, a significantly increased resistance to pentavalent antimony was observed. Similarly, increased amounts of HSP70 have been found in antimony-resistant clinical isolates of* L. donovani*. Noticeably, the resistant isolates contain increased levels (about 5-fold more protein) for both the cytoplasmic prototypical HSP70 and the mitochondrial HSP70 [161]. In another study with* L. donovani* clinical isolates, among the 12 overexpressed proteins in antimony resistant parasites, the cytosolic HSP70 and a membrane-associated fragment of HSP83/90 were identified [162]. Also, accumulation of HSP90 was previously observed in a study in which the proteomics of Sb(V)-sensitive and -resistant* L. donovani* strains, isolated from kala-azar patients, were compared [163]. Interestingly, overexpressing of HSP90 in a Sb-sensitive strain led to increased resistance to antimony and, noticeably, transfectants were also cross-resistant to miltefosine, another drug used for treatment of visceral leishmaniasis. In that work and in a previous article from the group [160], it has been suggested that HSP70 and HSP83 may interact with other proteins to negatively regulate the mitochondria-dependent apoptotic pathway that some drugs are activating in the parasite.

It has been suggested that antimonials kill cells by a process resembling programmed cell death (PCD) or apoptosis [164]. Current evidence also suggests that miltefosine treatment of* L. donovani* promastigotes leads to induce cellular alterations with features of metazoan apoptosis, including cell shrinkage, DNA fragmentation into oligonucleosome-sized fragments, and changes in membrane composition [165]. The increased expression of HSP90 in antimony resistant parasites has led to some authors to propose a role for HSP90 in protecting* Leishmania* from drug-induced PCD [163]. This hypothesis is based also on the fact that HSP90 and also HSP70 have been found to be a negative regulator of the mitochondrial cytochrome c-dependent apoptosis pathway in many cells and organisms [166]. Further support about the antiapoptotic properties of HSP90 has been obtained from studies in* L. donovani* parasites overexpressing histone H1. The level of HSP90 was found to decrease in promastigotes transfected with the histone H1 coding gene, and, interestingly, parasites overexpressing H1 were more susceptible to heat-shock and drug-induced apoptosis [167].

In certain regions of India, resistance to Sb(V) is so widespread that alternative drugs must be used as first choice. Amphotericin B (AmB) in its liposomal form is currently considered as the more effective drug available against visceral leishmaniasis, even though its high cost prevents their utilization in most affected countries [168]. Since some cases of drug resistance have been reported, studies intended to determine the AmB resistance mechanisms have been undertaken. Recently, a large-scale comparative proteomic study was carried out to identify proteins differentially expressed in an in vitro selected AmB resistant* L. infantum* line [169]. Many proteins involved in protein folding, such as heat-shock proteins and chaperonins, were found among the up-regulated proteins in this line, suggesting a putative role of these proteins in AmB resistance or tolerance. In particular, the heat shock proteins HSP90, HSP60, and cytoplasmic and mitochondrial members of HSP70 were found to be clearly upregulated in AmB resistant parasites.

In* L. major* lines resistant to the antifolate methotrexate, mitochondrial HSP70 (LmjF30.2470) and a type-3 HSP60 (LmjF36.2030) were found among the proteins more strongly overexpressed [170].

Increased expression of HSP70 mRNAs has been found in* L. donovani* amastigotes showing resistance to nelfinavir [171], an HIV-1 protease inhibitor, which was recently described as a powerful inhibitor of the intracellular growth of* Leishmania* in primary human monocyte-derived macrophages [172].

## 13. Concluding Remark and Future Tasks

We now have in hand most of the molecular chaperones existing in the different cellular compartments in* Leishmania*. The challenge for the future will be to understand how these distinct molecules work and how they are organized into functional networks to promote the life of this pathogen. Also, understanding of the mechanisms by which molecular chaperones detect stress and transduce signals is an important research field that requires further efforts. A rapid expansion in our knowledge about* Leishmania* biology will allow developing therapeutic strategies to combat leishmaniases.

## Figures and Tables

**Table 1:** Main classes of molecular chaperones.

Family name	Functions
HSP100	Unfolding, solubilisation of aggregates, proteolysis

HSP90	Protein maturation of steroid receptors, protein kinases, and other components of cellular signalling pathways. Organelle-specific variants exist

HSP70	Nascent-protein folding, refolding of denatured proteins, and translocation across membranes. Organelle-specific variants exist

HSP60/chaperonins	Protein folding and prevention of aggregation (bacterial and mitochondrial proteins)

CCT (TRiC)	Folding of cytoskeleton components

HSP40	Hsp70 ATPase activators and intrinsic chaperone activity

Small HSPs (sHSPs)	Prevention of aggregation, probable role in membrane homeostasis

**Table 2:** Members of the HSP70 family in *L*. *major* and their putative orthologs in humans.

*L*. *major* protein^1^ (GeneDB identifiers)	Human protein^2^ (UniProt ID)
HSP70 (LmjF.28.2770, LmjF28.2780)	Hsp70-1a/HSPA1A (P08107); Hsp70-1t/HSPA1L (P34931); HSPA6 (P17066)

	Hsp70-2/HSPA2 (P54652)

	Hsc70/HSPA8 (P11142)

BiP or Grp78 (LmjF.28.1200)	BiP/HSPA5 (P11021)

HSP70.c (LmjF28.2820)	

	Hsp70-14 (Q0VDF9)

MtHSP70-1 (LmjF30.2460–2490, LmjF30.2550)	MtHSP70/HSPA9 (P38646)

HSP70.4 (LmjF.26.1240)	

HSP70.a (LmjF.01.0640)	

HSP70.b (LmjF.26.0900)	

HSP110 (LmjF.18.1370)	Hsp110/HSPA4 (P34932)

HSP110 (LmjF.18.1370)	Hsp110-2/HSPA4L (O95757)

HSP110 (LmjF.18.1370)	Hsp105/HSPH1 (Q92598)

GRP170 (LmjF.35.4710)	Grp170/HSPH4 (B7Z2N4)

^1^Nomenclature according to Folgueira and Requena [15]. GeneDB accession numbers are shown in brackets.

^2^Nomenclature according to Kampinga and Craig [16]. UniProtKB accession numbers are shown in brackets.

**Table 3:** Classification of *L*. *major* HSP40s according to their structural features.

Name^1^	GeneDB ID	Size^2^	J domain	G/F-rich	Zn-finger	Type	Remarks
J1	LmjF32.3030	329	14–76	—	—	III	
J2	LmjF27.2400	396	6–70	78–94	120–205	I	
J3	LmjF21.0490	448	6–71	82–99	125–210	I	
J4	LmjF15.1220	478	6–71	87–115	144–227	I	
J5	LmjF36.1330	316	39–111	123–135	—	II	
J6	LmjF36.6270	345	4–70	93–125	—	II	GMPG repeats
J7	LmjF32.1940	323	3–71	78–105	—	II	
J8	LmjF24.0520	795	43–111	—	—	III	
J10	LmjF17.0460	216	36–103	139–166	—	II	
J11	LmjF04.0780	573	9–72	—	—	III	
J13	LmjF18.1490	184	14–93	—	—	III	
J14	LmjF08.0990	326	22–95/164–233	—	—	III	Two J domains
J15	LmjF19.0080	432	5–67	—	—	III	
J16	LmjF20.1130	653	137–202	210–242	—	II	SANT, DNA-binding domain (513–567) SMART accession number: SM00717
J17	LmjF12.1110	733	88–143	148–209	—	II	
J18	LmjF27.0410	321	16–80	—	—	III	
J19	LmjF34.4080	257	29–115	131–147	—	II	
J20	LmjF36.0610	261	172–239	—	—	III	J domain at C-terminus
J21	LmjF26.1410	536	405–474	—	—	III	J domain at C-terminus
J22	LmjF36.2110	286	19–83	111–124	—	II	
J23	LmjF18.0330	244	49–112	—	—	III	
J24	LmjF30.1790	456	27–89	134–167	—	II	
J25	LmjF26.1270	852	391–468	479–500	—	II	J domain in the middle
J26	LmjF17.0040	262	84–146	—	—	III	
J27	LmjF04.0940	487	86–150	156–210	247–325	I	
J28	LmjF26.1200	646	282–346	365–389	—	II	J domain in the middle
J29	LmjF24.1080	436	376–436	—	—	III	J domain at C-terminus
J30	LmjF07.0780	299	—	—	—	—	Truncated in *L*. *major *
J31	LmjF26.0940	843	36–106	—	—	IV	
J32	LmjF25.2190	377	8–74	80–119	322–351	I	
J33	LmjF36.4470	275	7–69	—	—	III	
J34	LmjF35.4630	491	134–203	202–240	—	II	
J35	LmjF14.0110	523	444–514	—	—	III	J domain at C-terminus
J36	LmjF25.1690	278	60–154	—	—	III	
J37	LmjF18.1430	1119	5–70	—	—	III	
J38	LmjF30.2450	336	15–69	—	—	III	
J40	LmjF10.1050	276	40–108	—	—	III	
J41	LmjF31.0510	596	283–348	360–391	—	II	J domain in the middle
J42	LmjF18.1650	580	518–580	—	—	III	J domain at C-end; Tetratricopeptide repeat domain (TPR, 136–463)
J43	LmjF35.4040	388	4–109	—	—	III	
J44	LmjF31.3100	217	18–89	—	—	II	
J45	LmjF32.3300	400	54–121	139–159	176–259	I	
J46	LmjF25.1100	395	54–123	142–149	174–257	I	
J47	LmjF20.0550	545	350–483	—	257–335	IV	J domain at C-terminus
J49	LmjF30.1030	416	52–119	—	—	III	
J50	LmjF35.2980	478	48–109	119–164	185–270	I	
J51	LmjF34.2430	808	700–770	775–807	—	II	J domain at C-end; two TPR domains (345–451 and 572–677)
J52	LmjF36.0500	510	377–448	455–510	—	II	J domain at C-end, 2 Tetratricopeptide repeat (TPR) domains: 17–118 and 254–359
J53	LmjF14.1330	564	431–503	513–560	—	II	J domain at C-terminus; two TPR domains (49–118 and 221–288)
J54	LmjF33.0900	580	3–66	85–140	—	II	
J55	LmjF28.1270	472	9–80	—	—	III	
J56	LmjF33.2690	267	45–136	—	—	III	
J57	LmjF29.1890	395	14–86	144–177	—	II	
J58	LmjF24.1300	807	15–87	—	—	III	
J59	LmjF30.2210	2458	1384–1463	—	—	III	putative endosomal trafficking protein RME-8
J60	LmjF09.1440	413	42–110	—	—	III	
J61	LmjF08.0650	286	3–78	—	—	III	
J62	LmjF34.0040	679	95–163	—	—	III	
J63	LmjF32.0590	316	40–107	—	—	III	
J64	LmjF07.0770	402	153–221	—	—	III	J domain in the middle
J65	LmjF34.3870	781	707–777	—	—	III	J domain at C-terminus; TPR domain (481–630)
J66	LmjF22.0080	331	156–279	—	52–139	IV	J domain at C-terminus
J67	LmjF36.0760	855	779–852	—	—	III	J domain at C-terminus; two TPR domains (237–338 and 574–645)
J68	LmjF24.1910	121	51–119	—	—	IV	
J69	LmjF36.4970	345	18–89	—	—	III	
J71	LmjF24.0070	439	49–109	—	—	III	
J72	LmjF35.3090	427	20–74	—	—	III	
J73	LmjF26.2520	489	29–86	—	—	III	
J74	LmjF28.1900	655	143–199	—	—	III	

^1^Nomenclature according to Folgueira and Requena [15].

^2^Number of amino acids deduced from the annotated gene sequences in the GeneDB database.
